# Where traditional Chinese medicine meets Western medicine in the prevention of rheumatoid arthritis

**DOI:** 10.1111/joim.13537

**Published:** 2022-07-19

**Authors:** Per‐Johan Jakobsson, Luke Robertson, Janika Welzel, Mingshu Zhang, Yang Zhihua, Gao Kaixin, Huang Runyue, Wen Zehuai, Marina Korotkova, Ulf Göransson

**Affiliations:** ^1^ Division of Rheumatology Department of Medicine Solna & Karolinska University Hospital Karolinska Institutet Stockholm Sweden; ^2^ Department of Pharmaceutical Biosciences Uppsala University Uppsala Sweden; ^3^ The Second Clinical Medical College Guangzhou University of Chinese Medicine Guangzhou China; ^4^ Section of Rheumatology and Immunology Research The Second Affiliated Hospital of Guangzhou University of Chinese Medicine (Guangdong Provincial Hospital of Chinese Medicine) Guangzhou China; ^5^ Key Unit of Methodology in Clinical Research Guangzhou University of Chinese Medicine (Guangdong Provincial Hospital of Chinese Medicine) Guangzhou China

**Keywords:** inflammation, rheumatoid arthritis, traditional Chinese medicine

## Abstract

Chinese medicine has a long tradition of use against rheumatoid arthritis (RA). The formulations are based on combinations of typically 5–10 plants, which are usually boiled and administered as a decoction or tea. There are few clinical trials performed so the clinical evidence is sparse. One fundamental of traditional medicine is to prevent disease. RA is an autoimmune, inflammatory and chronic disease that primarily affects the joints of 0.5%–1% of the population. In two out of three of the cases, the patients are characterised by the presence of autoantibodies such as the rheumatoid factor and the more disease‐specific autoantibody against citrullinated proteins, so‐called ‘ACPA’ (anticitrullinated protein/peptide antibodies). ACPA positivity is also strongly associated with specific variations in the HLA‐DRB1 gene, the shared epitope alleles. Together with smoking, these factors account for the major risks of developing RA. In this review, we will summarise the background using certain plant‐based formulations based on Chinese traditional medicine for the treatment and prevention of RA and the strategy we have taken to explore the mechanisms of action. We also summarise the major pathophysiological pathways related to RA and how these could be analysed. Finally, we summarise our ideas on how a clinical trial using Chinese herbal medicine to prevent RA could be conducted.

## Graphical abstract

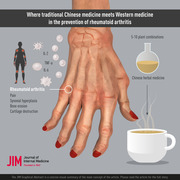

## Introduction

Where do our drugs come from? Today, the first response might be from big data, genomics and biotechnology; looking back a decade, the popular answer was likely from computer modelling and high‐throughput screening (HTS). Both answers are correct, but it remains a fact that more traditional routes to drug discovery are important even today. This review focuses on drug discovery from natural origin, and more specifically on how traditional medicine may still inspire the development of new drugs.

The earliest records of plants as medicines can be traced back nearly 5000 years to ancient Egypt and Mesopotamia, where plant products that remain important to this day (e.g., poppy latex, willow bark) were prescribed for the treatment of various ailments [1]. For thousands of years, plant‐based medicines formed the backbone of human healthcare all around the world. These medicines—and indeed, healthcare as a whole—underwent a great deal of development during this period, but it was not until the early 1800s that the active components of medicinal plants began to be purified. Among the first of these was morphine from poppy latex (*Papaver somniferum*) in 1806 and salicin (the precursor to acetylsalicylic acid) from willow bark (*Salix*) in 1828 [1, 2]. Both were soon sold in their pure form, marking the birth of the modern pharmaceutical industry.

**Fig. 1:**
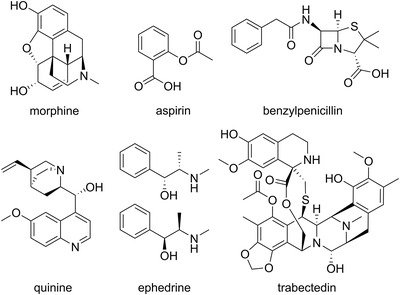
Some famous natural product drugs. Morphine, acetylsalicylic acid, quinine and ephedrine are plant natural products, benzylpenicillin is a fungal metabolite and trabectedin was discovered from a marine invertebrate.

Since then, Nature has been shown to be a reliable source for the development of new drugs, providing us with blockbusters such as penicillin, quinine, lovastatin, vincristine/vinblastine and paclitaxel (Fig. 1). More recent examples include exenatide (2005), trabectedin (2007) and plitidepsin (2018) [3]. Some of these were discovered by serendipity, some by large screening campaigns whereas others have arisen from their use in traditional medicine.

Traditional medicine remains a key part of healthcare across the world. Whether or not one trusts its efficacy, its influence cannot be ignored: in its latest account, the World Health Organization reports that 170 (88%) of its member states have national policies for traditional and complementary medicine [4]. In some regions, traditional medicine is the only healthcare option available, with its use based on the trust of individual healers or medicine men and women. In others, it is built into the healthcare system and used in parallel with modern pharmaceuticals. The latter is the case for much of traditional Chinese medicine (TCM), and this opens a window of opportunity to include clinical data as a starting point for drug discovery.

The prime example of drug discovery from TCM is that of the antimalarial drug artemisinin from sweet wormwood (*Artemisia annua*). This compound has saved millions of lives to date, and its discovery was rewarded with the Nobel Prize in Physiology or Medicine to Tu Youyou in 2015 [1, 5]. However, its path from traditional medicine to a modern drug was less than straightforward, and may serve to highlight caveats along the way: how can one correlate traditional information with clinical symptoms of disease? Which chemical methods should be used to extract and isolate any pure compound responsible for activity, and which bioassays are suitable to detect the active compound(s) and determine their mechanism(s) of action? In this review, we address those questions from the viewpoint of rheumatoid arthritis (RA).

## Rheumatoid arthritis

RA is a chronic autoimmune inflammatory disorder mainly characterised by synovial joint inflammation and progressive destruction followed by disability. The disease is associated with important comorbidities, reduced patient life expectancy and heavy economic burden. RA affects 0.5%–1.0% of the Western and 0.2%–0.9% of the Chinese populations and is more prevalent in women [6, 7]. Autoantibodies that target various molecules including post‐translationally modified self‐epitopes (e.g., rheumatoid factor [RF] and anticitrullinated protein/peptide antibodies [ACPA]) have been found in two thirds of RA patients and can be detected years before the onset of the disease [8]. The gene–environmental interaction between HLA‐DRB1 alleles encoding the shared epitope and smoking is the strongest risk for developing ACPA‐positive RA [9].

**Fig. 2:**
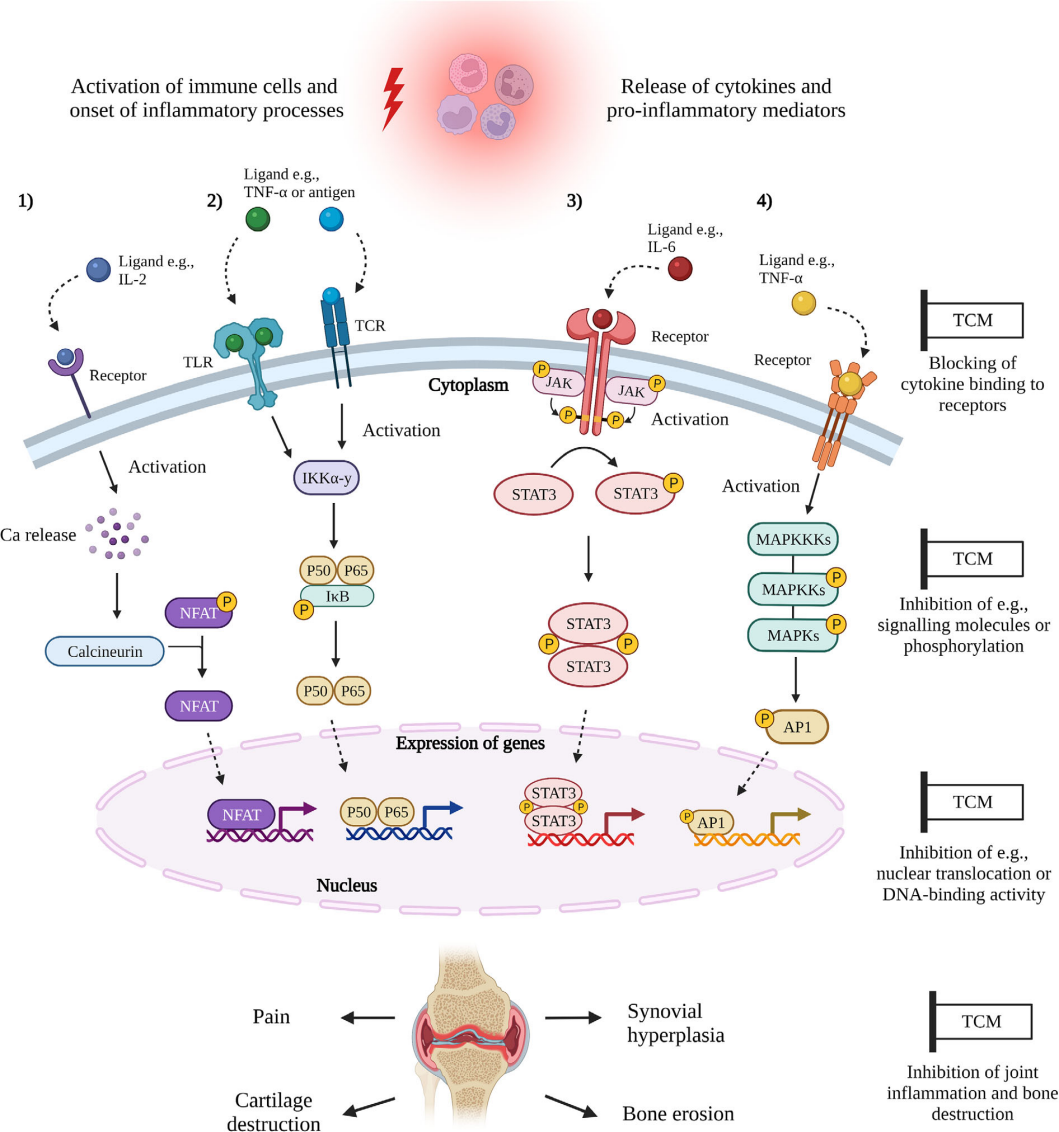
Impact of herbal medicine on rheumatoid arthritis (RA) pathways. Several immune cell types are recruited and activated in inflamed joints during the RA disease course and initiate the release of pro‐inflammatory mediators and cytokines. Those mediators bind to specific receptors on immune cells and further activate intracellular signalling pathways. Four of the main pathways implicated in RA are displayed in this figure. (a) After binding the ligand to its specific receptor, the nuclear factor of activated T cell (NFAT) pathway is activated through calcium/calcineurin release. (b) Binding of specific ligands to toll‐like receptors (TLR) or T‐cell receptors (TCR) activates the NF‐κB signalling pathway. The inhibitor of ĸB kinase (IKK) complex is activated, resulting in phosphorylation of the IĸB and release of the NF‐κB p50/p65 complex that translocates into the nucleus. (c) Multiple ligands bind their specific receptors and activate the JAK/STAT pathway. Activation of Janus kinase (JAK) leads to phosphorylation of signal transducer and activator of transcription (STAT) molecules, which dimerise and translocate into the nucleus. (d) The induction of MAPK pathway activates several kinases that regulate downstream transcription factors, increasing the expression of transcription factor AP‐1. The transcription factors mentioned above bind to specific response elements on the DNA, thus activating the expression of a variety of genes involved in inflammatory processes. Traditional Chinese medicine (TCM) can affect the signalling pathways by blocking the binding of mediators to their receptors, the activity of kinases and phosphorylation processes, the nuclear translocation of the transcription factors and the DNA binding to their response elements. The expression and activation of immune‐modulatory genes lead to synovial hyperplasia, bone erosion and cartilage destruction. TCM can inhibit those inflammatory responses as well as joint destruction.

In RA joints, there is a complex network of activated T and B lymphocytes, synovial fibroblasts, macrophages, neutrophils and other cells that maintain the production of numerous cytokines, chemokines, metalloproteinases (MMPs), growth factors and lipid mediators amplifying and driving the inflammatory and destructive processes into chronicity [7, 10]. Many pro‐inflammatory mediators bind specific receptors and activate intracellular signalling pathways leading to changes in the expression of genes involved in inflammatory and destructive processes in the rheumatic joint (Fig. 2). The aims of the current treatment for RA patients are to suppress inflammatory disease, achieve disease remission, prevent progressive joint damage and preserve joint function. The development of biological disease‐modifying antirheumatic drugs (bDMARDs) and targeted synthetic DMARDs (tsDMARDs) has revolutionised the management of RA, especially in patients with insufficient response to methotrexate (MTX) and other treatments [11]. bDMARDs are potent immunomodulatory agents that specifically antagonise inflammatory cytokines (TNF‐blockers, tocilizumab), target T lymphocyte costimulation (abatacept) or deplete B lymphocytes (rituximab). tsDMARDs are oral small molecule drugs that selectively target intracellular signalling pathways and modulate immune and inflammatory responses. Four inhibitors of the JAK/STAT signalling pathway (tofacitinib, baricitinib, upadacitinib and filgotinib) have been approved for the RA treatment and many other small molecular inhibitors are under development [12].

Despite the fact that both bDMARDs and tsDMARDs are effective in RA treatment by suppressing disease activity and joint destruction, these therapeutic approaches demonstrate ‘therapeutic ceiling’ effects, with 10%–20% of patients not achieving remission [7, 13, 14]. Furthermore, 5%–10% of RA patients are not responsive to several treatments and demonstrate a ‘difficult‐to‐treat’ pattern with persistent symptoms [15]. The phenomenon of ‘therapeutic ceiling’ and treatment‐refractory RA indicates that important pathogenic mechanisms remain untargeted and that there is a remaining clinical void to fill in the treatment of RA.

The inability of patients to respond to therapeutics with different modes of action can be explained by the heterogeneity of immunologic mechanisms and pathogenic pathways in RA [15, 16]. Several RA synovial phenotypes with distinct cellular composition (myeloid–lymphoid–fibroid phenotypes), cell infiltration (diffuse‐follicular), signalling pathways (NF κB–JAK/STAT–Wnt or TGF‐β pathways) and molecular signatures have been described [17, 18, 19]. The particular synovial phenotype may require specific treatment targeting relevant molecular mechanisms and signalling pathways. Indeed, good clinical response to TNF‐blockers was observed in RA patients with myeloid synovial phenotype, whereas the clinical response to IL‐6 receptor blocker tocilizumab was detected in the lymphoid synovial phenotype [17]. Another approach to improve responsiveness to treatment is to suppress ‘nonimmune’ players in RA, such as synovial fibroblasts and neutrophils. These cells greatly promote inflammatory and destructive processes in the synovium and are suggested as promising therapeutic targets in RA [13, 20, 21].

## Early treatment and prevention of RA

The basis for the prevention of RA lies in the fact that autoantibodies precede the onset of disease by years, the beginning of unspecific musculoskeletal symptoms such as arthralgia and fatigue, and exposure to environmental risks like smoking. These individuals are considered ‘at risk’ for developing RA.

The 2010 ACR/EULARs classification criteria [22] were designed to catch the RA diagnosis at an earlier stage than the 1987 ACR criteria. For instance, if an individual presents with one instance of clinical arthritis in one defined finger joint, high titre of ACPA (greater than three times the reference value) and increased acute phase reactions, the individual would be classified as having RA. The current ‘at risk’ cohorts include individuals as described above (but without clinical arthritis) and they are followed over time [23]. In 1 year (depending on the stringency of inclusion criteria), as many as 50% may develop their first arthritis and thereby fulfil the 2010 RA classifications criteria. The realization that one can identify individuals with such high risks of developing RA enables studies of very early treatments (it is well established that early treatment enables long‐lasting remission) as well as reaching toward prevention. Several prevention trials have been reported and are ongoing [23]. The first trial attempting to prevent RA demonstrated the possibility to delay the onset of RA by rituximab treatment once [24]. Abatacept was also recently shown to significantly delay/prevent the onset of disease [25]. But what would be the most appropriate drug for the prevention of RA? Induction of tolerance is one obvious track, but so far, has not been successful. An ideal drug or composition of drugs would ‘calm down’ the immune system and support the resolution of inflammation. A preventive drug should be safe with no severe side effects; it should also be at low cost to be used globally and, importantly, have evidence for efficacy and mechanisms of action.

## RA pathways

Recently, research focus has switched to intracellular signalling pathways as possible therapeutic targets in RA, and tsDMARDs have become a part of the therapeutic arsenal in RA [26]. Importantly, tsDMARDs suppress intracellular signalling pathways and regulate both the action and the production of a range of cytokines. The main intracellular signalling pathways that are implicated in the pathogenesis of RA include the Janus Kinase/signal transducers and activators of transcription (JAK/STAT), Nuclear factor kappa B (NF‐κB), the mitogen‐activated protein kinases (MAPK) and phosphatidylinositide‐3 kinase/protein kinase B (PI‐3K/AKT) [27, 28, 29]. In addition, other pathways such as the nuclear factor of activated T cells (NFAT), spleen tyrosine kinase (Syk), Bruton's tyrosine kinase (Btk), the Notch signalling and the Wnt signalling have also been considered therapeutic targets in RA [29, 30, 31, 32, 33]. There are also intracellular interactions between the major signalling pathways resulting in a complex regulatory network that contributes to sustained inflammation. Below, we describe several interesting signalling pathways in RA that are highly relevant as drug targets (Fig. 2).

The JAK/STAT pathway is a central signalling cascade in inflammatory, immune and cancer cells that operates downstream of the type I and type II cytokine receptor superfamily [26]. In RA, the JAK/STAT pathway plays a critical role in the disease progression, transmitting signals from a plethora of cytokines, interferons and growth factors to induce different effector genes [34]. The activation of this pathway leads to leukocyte recruitment and production of pro‐inflammatory cytokines, chemokines, MMPs and vascular endothelial growth factor (VEGF), perpetuating a chronic cycle of inflammation and joint destruction [34, 35, 36]. In RA patients, JAK/STAT inhibitors have demonstrated both significant clinical efficiency and a safety profile comparable to TNF inhibitors. JAK/STAT inhibitors delay radiographic progression and efficiently relieve pain [12].

The NF‐κB signalling pathway is another key inflammatory pathway in RA, which is rapidly activated in immune and inflammatory cells by various inflammatory cytokines and via pattern‐recognition receptors and T‐cell and B‐cell receptors [37, 38]. NF‐κB activation leads to the expression of genes that control cell proliferation, apoptosis, angiogenesis, production of autoantibodies, inflammatory cytokines, chemokines and MMPs, and greatly contributes to inflammation and joint destruction in RA [39, 40]. Numerous inhibitors have been designed to efficiently block distinct steps of NF‐κB signalling, including inhibitors of IKK activity, proteasome, nuclear translocation and DNA binding activity; however, there are still no clinically available selective NF‐κB‐based drugs for RA treatment [40, 41].

MAPK is a family of serine/threonine protein kinases that include the extracellular signal‐regulated kinases (ERKs), c‐jun N‐terminal kinase (JNK) and p38 MAPK. MAPKs phosphorylate a variety of intracellular proteins, including transcription factors that consequently regulate the expression of genes involved in cell proliferation, differentiation, survival, cellular stress and inflammatory responses. MAPKs contribute to the production of the inflammatory cytokines, chemokines, prostaglandins, MMPs and growth factors involved in joint inflammation and destruction and are important players in the RA pathogenesis [42, 43, 44]. The therapeutic potential of MAPKs as drug targets has been clearly demonstrated using specific inhibitors in experimental models of arthritis [45, 46, 47]. However, the development of MAPK inhibitors has been hindered by selectivity and toxicity issues [44, 48].

Exposure to pro‐inflammatory cytokines, growth factors and antigen stimulation of T‐cell and B‐cell receptors activate the NFAT signalling pathways in RA [49, 50]. This important signalling pathway controls the activation of macrophages, synovial fibroblasts, chondrocytes, as well as B‐ and T‐cell activation and differentiation [30, 49, 50]. The NFAT pathway contributes to chronic inflammation, stimulating the production of pro‐inflammatory cytokines, chemokines and MMPs; angiogenesis and osteoclastogenesis [30].

Therefore, these pathways are all relevant for the development of bioassays to explore the functions of plant‐based drugs in a high‐throughput fashion. Indeed, several patented TCM drugs (e.g., a ready‐made capsule or tablet), formulations (e.g., decoctions of plants) and isolated compounds from plants have shown an effect on RA pathogenesis in general, and these signalling pathways in particular [51, 52, 53].

## Impact of herbal medicine on RA pathways

There are numerous reports of effects of plant preparations and compounds on the JAK/STAT pathway. This includes several isolated compounds, which reduce either kinase activation or phosphorylation level in vivo or in vitro. For example, the alkaloid sinomenine from *Caulis sinomenii* inhibits JAK, as confirmed by the regulation of cytokine levels (e.g., IL‐17 and TNF‐α) and reduction of JAK/STAT phosphorylation [54]. Similar effects have been shown for decoctions tested in a clinical trial, demonstrating JAK/STAT inhibition activity [55] and for a patented Chinese medicine, which decreased JAK/STAT phosphorylation and ameliorated joint symptoms in collagen‐induced arthritis (CIA) rats [56, 57].

Several TCM formulas have been described with NF‐κB‐regulating activity in animal models (adjuvant‐induced arthritis (AIA) and CIA rats). Mechanisms include the reduction of cytokine levels (e.g., IL‐1β, IL‐6, IL‐8, TNF‐α, IFNγ) by either stimulating Nrf2 expression or inhibiting NF‐κB phosphorylation [58, 59]. Plant‐derived compounds that have shown effects on these pathways include curcumin isolated from *Curcuma longa*, which inhibits TLR2, MyD88 and NF‐κB p65 expression in the CIA rat model, and NF‐κB activation by interfering with the phosphorylation and degradation of IκB‐α [60, 61, 62, 63]. There are several other examples, such as flavonoids (e.g., baicalin), terpenes (e.g., paeoniflorin) and alkaloids (e.g., piperlongumine) [64].

Other formulations and compounds target MAPK. For example, a formulation containing four classic anti‐inflammatory plants reduces levels of IL‐6, IL‐8 and TNF‐α by regulating both PI3K/AKT and Ras/MAPK pathways [65]. A combined decoction of *Paeonia lactiflora* and *Atractylodes macrocephala* has been shown to downregulate mRNA expression of iNOS, TNF‐α, IL‐6 and MCP‐1 by the MAPK pathway [66]. Examples of pure compounds include the polyphenols chebulagic acid and chebulanin isolated from *Terminalia chebula*, which have been shown to inhibit the MAPK pathway in lipopolysaccharide (LPS)‐induced RAW 264.7 macrophages. Chebulanin also reduces IL‐6 and TNF‐α levels and interferes with phosphorylation of MAPK p38 and JNK in the CIA model [67].

## Example of a decoction currently under investigation

Despite numerous reports on activity in different pathways and widespread use of TCM in RA treatment, there are a limited number of clinical investigations for treatment of RA. So what is the best starting point for studies, and how should one approach the selection of a formulation or plants to study? As an example of the further discussions, we chose the formulation here referred to as decoction A (DA), which is based on a long clinical practice in China. DA is composed of eight plants (Table 1) with a long history of use to treat RA. However, the specific combination of plants in DA is new. DA has gone through animal experiments and a pilot clinical observation study, which both indicated that it has potential in reducing the progression towards RA (unpublished results). At such an outset, chemical, pharmacological and clinical studies can now be performed.

**Table 1:** Contents of the herbal formulation decoction A (DA). DA is a combination of two old formulas from the books Synopsis of Golden Chamber and Qi Xiao Liang Fang (authors’ translation: ‘Special and Marvelous Formulas’). The Chinese names of these two formulas are Huangqi‐Guizhi‐Wuwu Decoction and Yiyiren Decoction [91, 92]

Latin name	Chinese name	Pinyin	Plant part	Dose (grams)
*Coix lacryma‐jobi*	薏苡仁	Yiyiren	Seed	30
*Angelica sinensis*	当归	Danggui	Root	10
*Paeonia lactiflora*	白芍	Baishao	Root	10
*Ephedra sinica*	麻黄	Mahuang	Stem	3
*Cinnamomum cassia*	桂枝	Guizhi	Stem	10
*Astragalus mongholicus*	黄芪	Huangqi	Root	30
*Atractylodes lancea*	苍术	Cangzhu	Root	15
*Glycyrrhiza uralensis*	炙甘草	Zhigancao	Root	15

## Identification of bioactive compounds in DA

A major challenge of DA (as with any other traditional plant‐based medicine) is its chemical complexity. Identifying important biologically active constituents from a single plant (which may produce thousands of compounds at detectable concentrations) is already very difficult. In the Chinese medicine clinic, all eight plants are extracted (by decoction) and administered together as a ‘tea’ or dry formulation. This means that the final product represents an unusually high chemical complexity. It also raises several important questions: if the formulation is effective, how does it work? Are there specific compound(s) that are responsible for its effectiveness, or is it reliant on multiple compounds or plants acting together synergistically?

To answer these questions, formulation DA is first simplified to study the biological activity of each of the eight plants on their own. Plants that show good biological activity can be separated further using chromatographic methods and then tested for biological activity again. This process is called bioassay‐guided purification and it allows for the comparison of biological activity shown by the herbal teas to that of individual plants and, ultimately, purified compounds. The methods used for the characterization of DA were adapted from the workflow currently in use at the US National Cancer Institute Program for Natural Products Discovery [68, 69, 70]. The process involves three major steps: (1) extraction, (2) purification and (3) structural characterization.

### Extraction

**Fig. 3:**
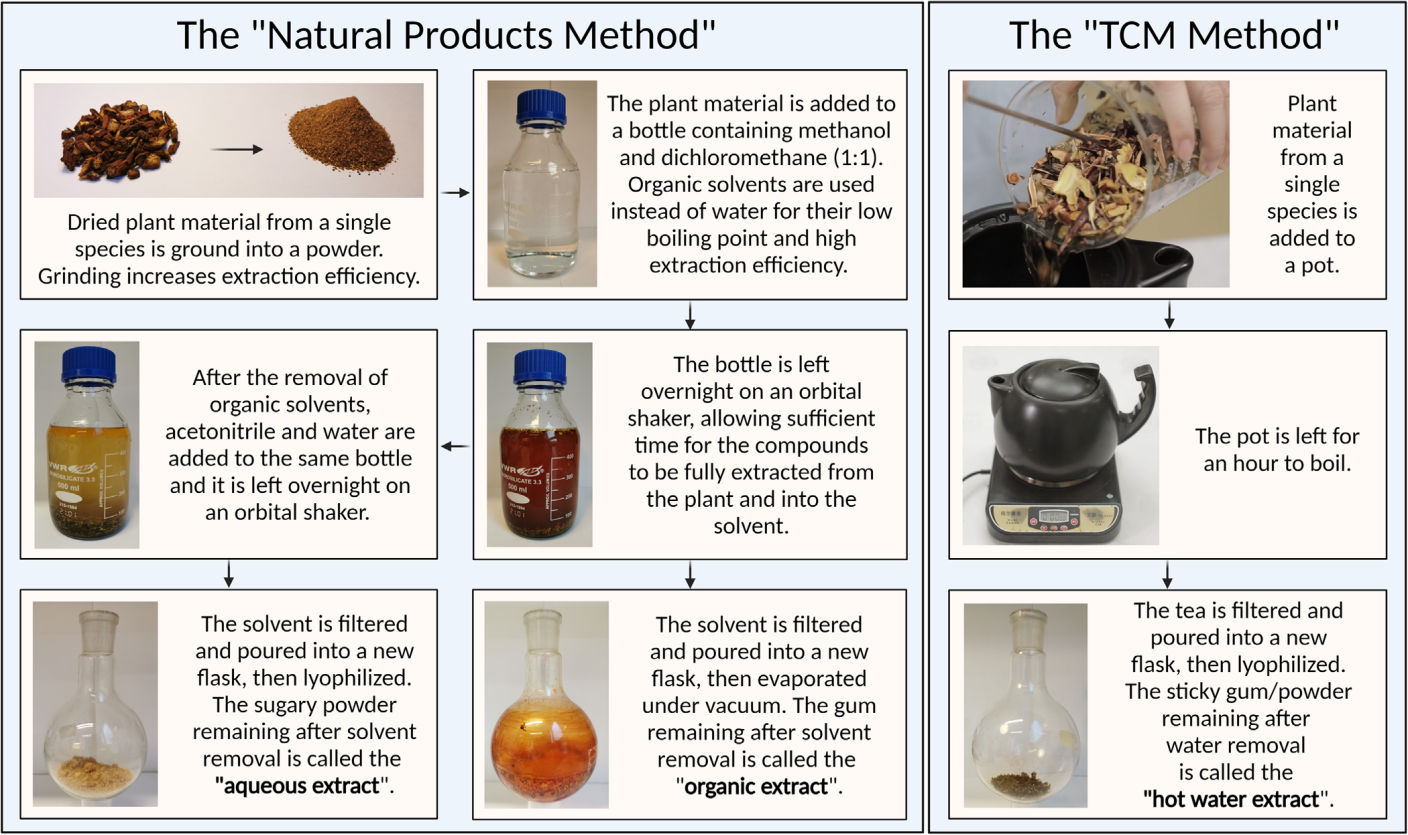
An overview of the two extraction methods used within the project. The ‘natural products method’ is based on typical chemical approaches for the isolation of biologically active substances from natural sources, whereas the ‘TCM method’ (where TCM refers to traditional Chinese medicine) is used to create a product that can be more directly compared to herbal teas.

**Fig. 4:**
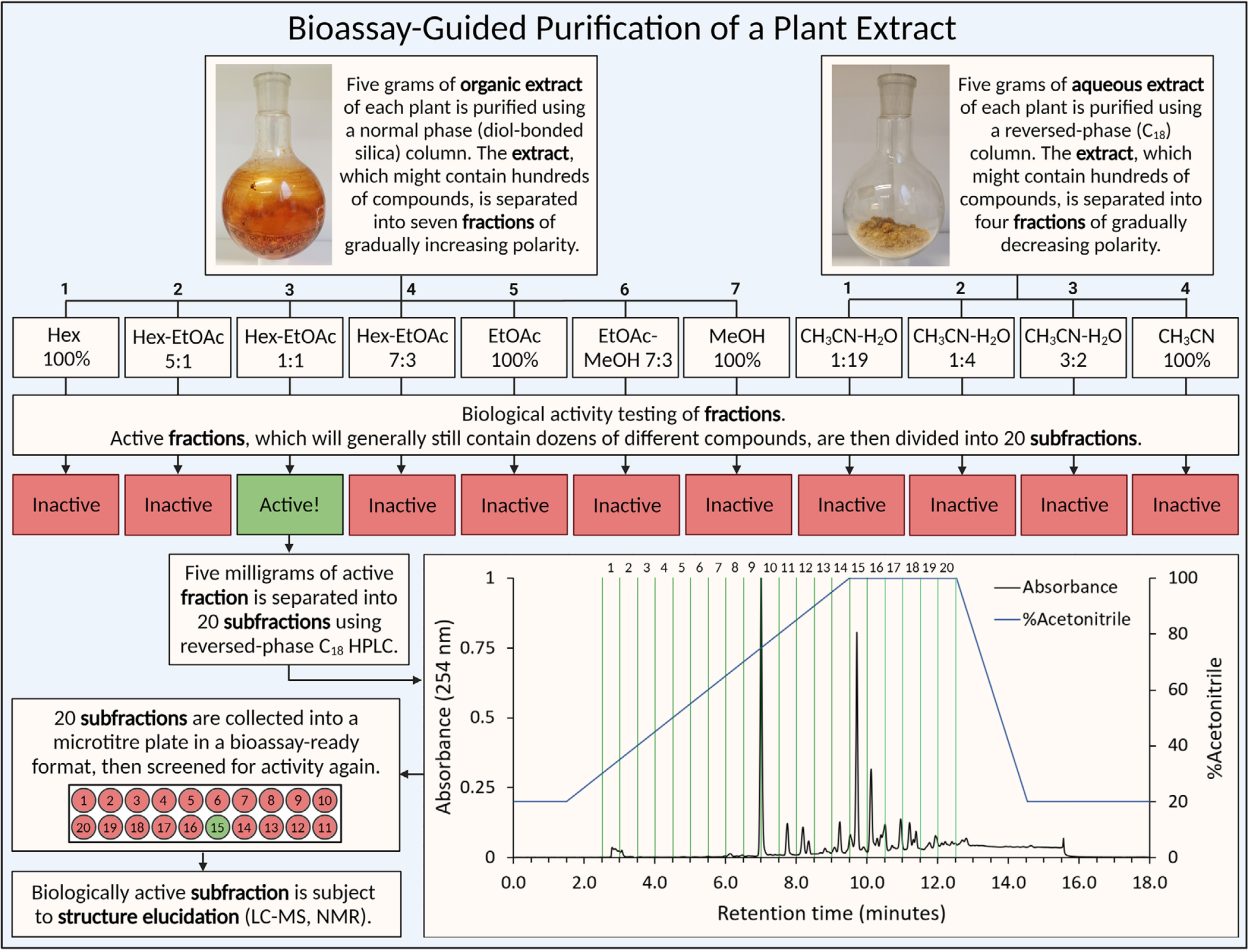
The workflow is used to isolate a biologically active compound from a plant. Extracts are the first subject to a crude ‘stepwise’ fractionation process, dividing them into four or seven fractions. After biological activity testing, fractions are further purified using reversed‐phase high‐performance liquid chromatography and screened again. EtOAc, ethyl acetate; Hex, hexane; MeOH, methanol.

The process begins with dried plant material. In this step, a plant is extracted by one of two methods: one ‘natural products’ method and one ‘TCM method’ (Fig. 3). In the ‘natural products’ method, dried plant material is ground into a powder and then macerated in organic solvents (dichloromethane and methanol) overnight, before being decanted, filtered and evaporated. This residue is labelled as the ‘organic extract’. The extraction solvent, dichloromethane:methanol (1:1), can effectively extract a wide range of compounds, ranging from nonpolar to polar [68]. After the removal of organic solvents, the plant material is soaked again in water:acetonitrile (2:3) overnight, then the solvent removed as above. This part is then labelled the ‘aqueous extract’. This second extraction, which utilises a more polar solvent system, is performed to ensure extraction of more polar compounds that may have been missed in the organic extraction. Finally, in the ‘TCM method’, new plant material is extracted by decoction (boiling in water), and the solvent decanted, filtered and lyophilised. This is called the ‘hot water extract’ and should be more similar to the formulation consumed by patients. All three extracts are analysed using liquid chromatography–mass spectrometry (LC‐MS) and liquid chromatography–ultraviolet spectroscopy to obtain spectral fingerprints, then subjected to biological assays (Fig. 4). However, since all three are very chemically complicated, they must be purified further using chromatographic methods to identify specific biologically active compounds that could potentially be developed into new drugs.

### Purification

The first step in the purification process is normal‐phase chromatography (Fig. 4). In this step, the organic extract from each plant is divided into seven fractions using a diol‐bonded silica column [69]. A three‐solvent system of gradually increasing polarity is used, consisting of hexane (nonpolar), ethyl acetate (medium polar) and methanol (polar). The first fractions (1–2) mostly contain lipids, while the later fractions (6–7) are rich in sugars. Since both are unlikely to make good drugs, their separation from the more chemically appealing medium polarity compounds localised in fractions 3–5 is desirable. The aqueous extract of each plant is also purified, using reversed‐phase (RP) (C18) chromatography and stepwise elution to give four additional fractions. The major problem with the aqueous extract is the huge amount of sugar, which may constitute 80%–90%+ of its total mass, potentially drowning out more ‘drug‐like’ compounds present in lower concentrations. By eluting the column with solvents of decreasing polarity, sugars can be concentrated into the first fraction, allowing the more interesting medium‐polarity compounds to be distributed across the other three.

Each of the 11 fractions (seven organic, four aqueous) are then tested for biological activity. Since these are unlikely to contain pure compounds, all active fractions are further purified using RP high‐performance liquid chromatography (HPLC) [70]. This is done using a solvent gradient from 20% to 100% acetonitrile over 12.5 min on a semipreparative C18 column. From each fraction, 20 ‘subfractions’ are collected at 30‐s intervals directly into a 96‐well microtitre plate. Subfractions are then tested for biological activity and positive hits are analysed using LC‐MS and nuclear magnetic resonance (NMR) spectroscopy (Fig. 4).

### Structural characterization

**Fig. 5:**
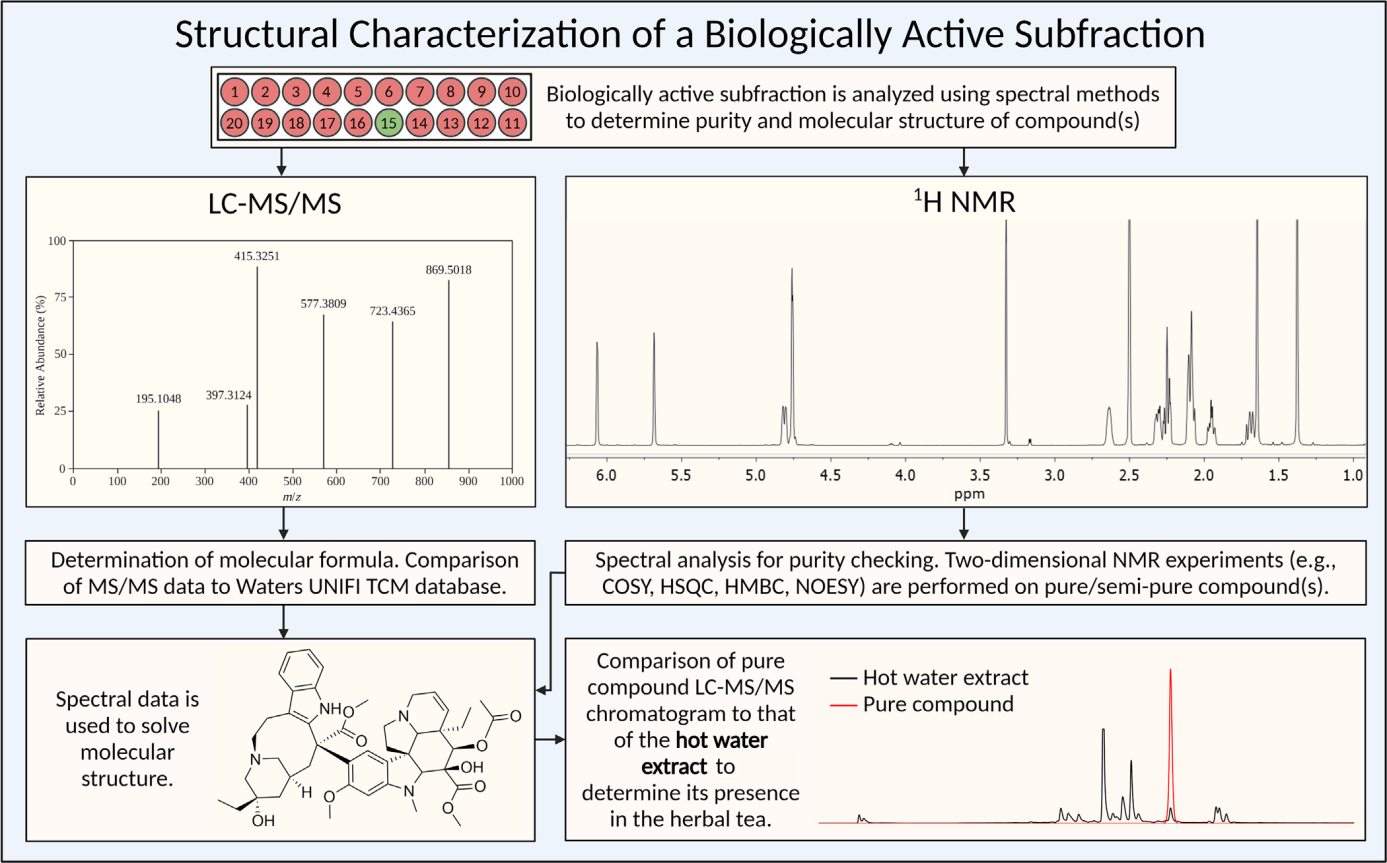
Methods used to determine the structures of biologically active compounds.

With a biologically active subfraction in hand, an assessment of its purity and molecular contents is undertaken (Fig. 5). An aliquot of the subfraction is first analysed with high‐resolution LC‐MS/MS. From this, a basic evaluation of purity is made, and an MS/MS fingerprint of the active compound(s) is acquired. This data can then be imported into Waters UNIFI™ software and compared to its in‐built TCM library, which contains MS/MS fragmentation data of thousands of compounds found in common TCM plants. A structural proposal of the compound is offered, although this offers varying degrees of accuracy. To unequivocally establish the structure of the active compound(s), each active subfraction is analysed by 1H NMR. Subfractions deemed to be of adequate purity are then analysed with two‐dimensional NMR experiments. If the subfraction contains insufficient material for NMR analysis, a scaled‐up HPLC of the active fraction is performed, as described by Grkovic et al. [70]. Interpretation of this data results in the determination of the molecular structure of the biologically active compound and marks the end of the bioassay‐guided purification process.

Results from chemical and pharmacological characterisation are incorporated into the workflow described above and to complement existing databases such as the Dictionary of Natural Products [71]. This is important not the least for subsequent LC‐MS/MS analyses of the active compound in the ‘hot water extract’ from the same plant species. This is done to determine whether the isolated compound is actually present within the herbal tea prepared by patients (Fig. 5).

## In vitro bioassays

To investigate the earlier described relevant signalling pathways activated in RA and to be able to determine active ‘hit’ fractions and subfractions, it is important to implement adequate bioassays. There are several common HTS assay formats, including targeted (biochemical, binding‐based) and phenotypic (cell‐based) approaches [72, 73]. Phenotypic screening has been successfully implemented in drug discovery efforts due to its higher probability to predict in vivo effects. The most commonly used bioassays include cell viability assays, signalling pathway assays and disease‐related phenotypic assays [73].

**Fig. 6:**
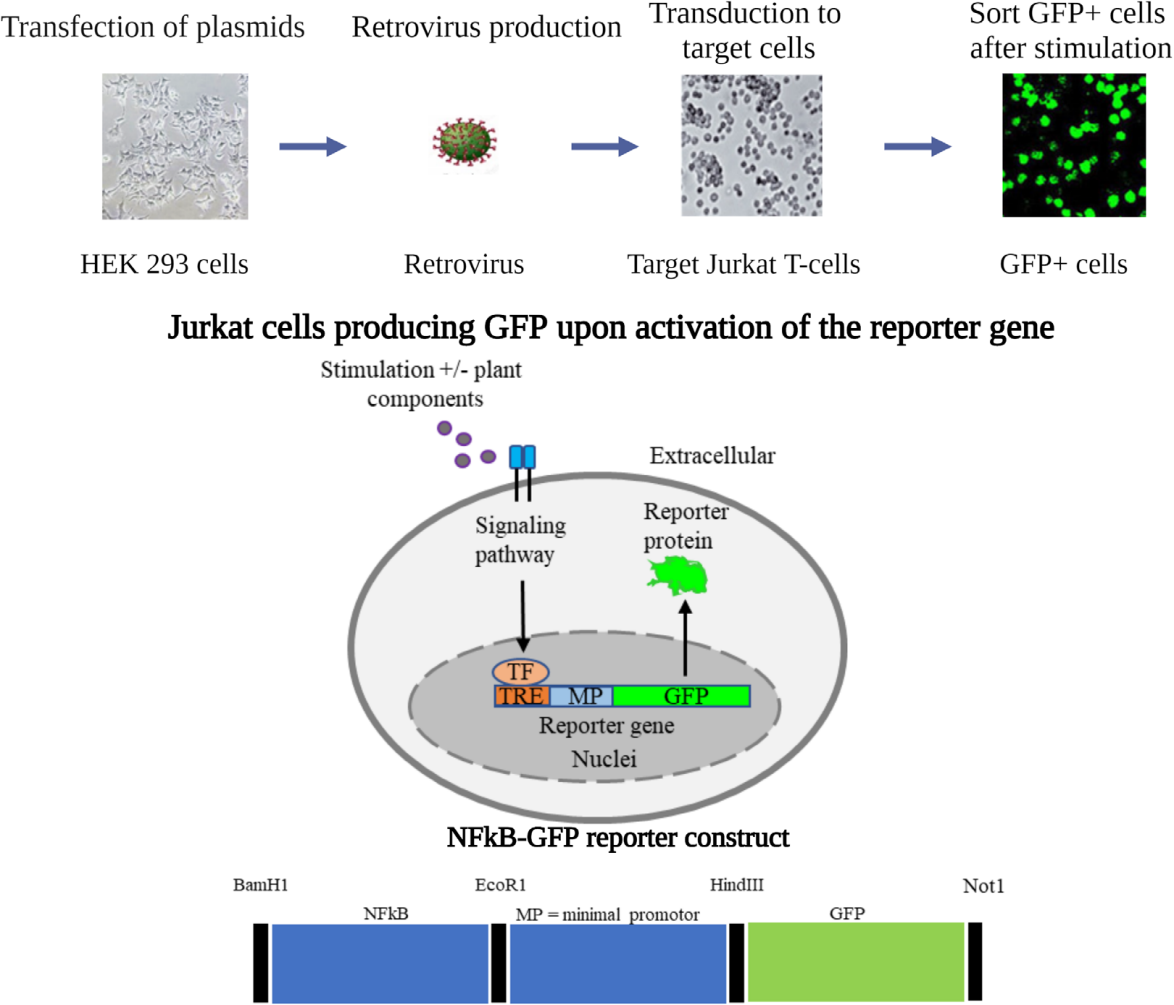
Example of the generation of a NF‐κB reporter assay. Here, HEK‐293 cells are transfected with the expression plasmid (NF‐κB‐GFP) and a packaging plasmid for retrovirus. After 48 h, the cells have produced full virus particles with the gene of interest inserted in their genome. After collection of viruses from the supernatant, they are transduced to the target cells (here exemplified by Jurkat T cells). The sequence of the gene of interest contains long terminal repeat regions, which are recognised by enzymes of the target cells and randomly inserted into their genome. When the pathway is activated, transcription factors (TF) will bind to the transcriptional response element (TRE) of NF‐κB and hence activate the expression of GFP.

To study the effects of DA, reporter gene assays covering the most relevant pathways (NF‐κB, NFAT, STAT3, and STAT5) were developed. In these reporter cell assays, green fluorescent protein (GFP) is under the regulation of the specific pathway and thus expressed as the grade of activation of the respective pathway (Fig. 6). These assays provide a robust and efficient method for screening in a medium‐throughput format, and to detect extracts and fractions that impact the activity of the studied pathways. In combination with the use of different cell lines for example, T cells, B cells, monocytic or disease‐related phenotypic cells (e.g., synovial fibroblasts), these assays reflect various immune processes of RA pathogenesis.

**Fig. 7:**
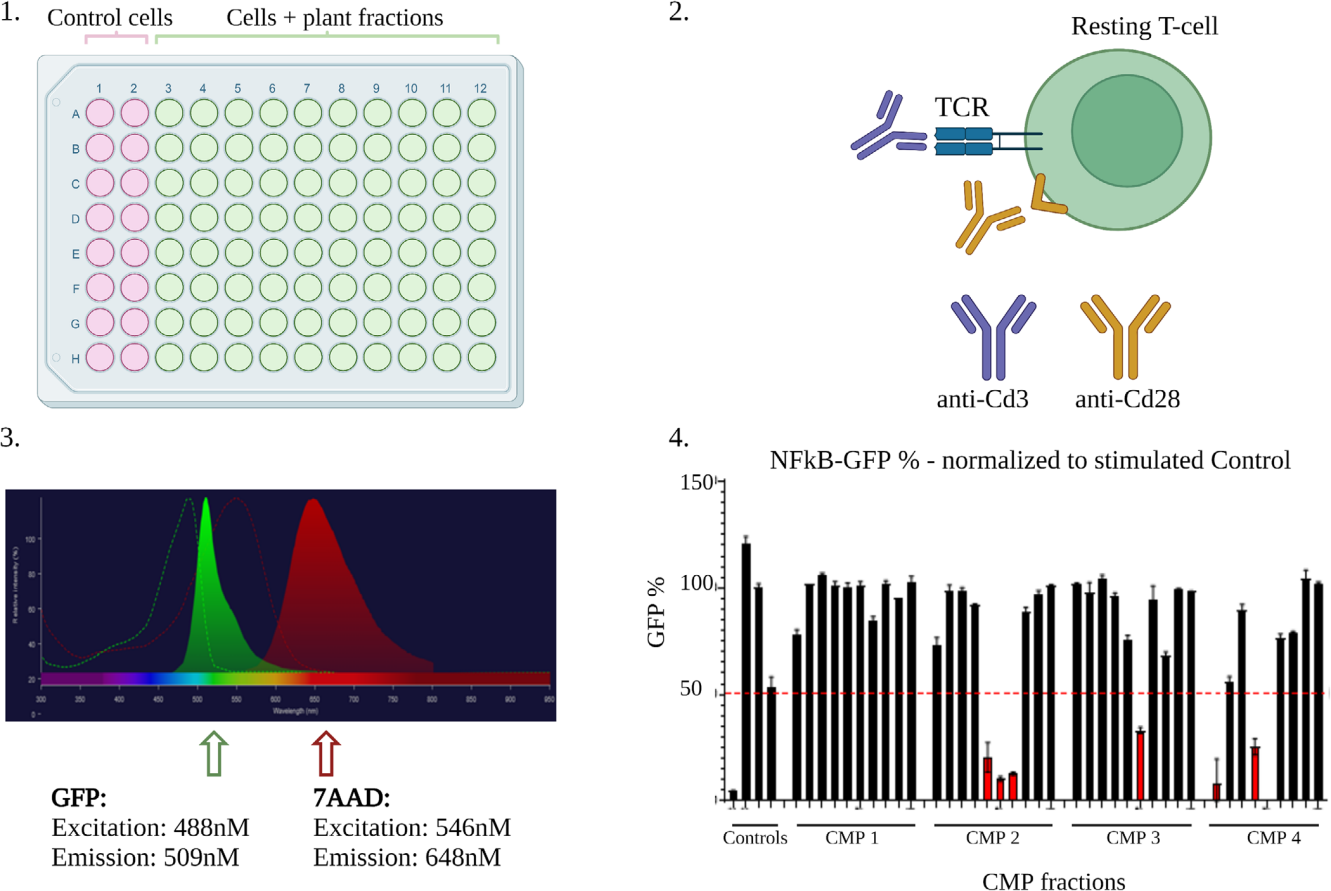
Screening of Chinese medicinal plant (CMP) fractions and pure compounds. In the first step, cells are seeded in 96‐well plate format for medium throughput screening (a). Following that, the cells are stimulated with the antibodies anti‐Cd3 and anti‐Cd28, which act through activation of the T‐cell receptor (TCR) (b). This leads to an upregulation of the NF‐κB signalling pathway. After incubating for 24 h with the fractions or pure compounds, the cell viability and GFP levels can be measured with flow cytometry (c). To evaluate the cell viability, a fluorescent dye for DNA staining is used (7‐aminoactinomycin D [7‐AAD]). To evaluate the pathway activity, the fluorescence of GFP is measured. The fluorescence of both markers can be analysed simultaneously since their emission wavelengths differ significantly (c). Only single and viable cells are considered in the analysis of the GFP expression in order to identify fractions and compounds with eminent bioactivities (d).

In the example of the Jurkat T‐cell assays, anti‐CD3 and anti‐CD28 are used as activators of the T‐cell receptor (TCR) to stimulate cells and activate respective pathways. Flow cytometry is used as a read out, which enables the evaluation of cell viability simultaneously with the reporter protein GFP (Fig. 7). Controls, for example, commercially available inhibitors of the pathway(s), should always be included.

In addition to signalling pathway analyses, one may consider screening the effects of DA and fractions thereof on the biosynthesis of effector molecules derived from the innate immune system. For instance, the arachidonic acid cascade has important roles in the inflammatory aspects of RA. Indeed, enzymes and receptors involved in the biosynthesis and actions of arachidonic acid–derived bioactive lipids, such as prostaglandins and leukotrienes, are key therapeutic targets [74]. The main, nonsteroidal anti‐inflammatory drugs target the prostaglandin cascade by inhibition of the cyclooxygenase I/II and are the most widely used drugs when it comes to the treatment of inflammation and pain [75]. To specifically target inflammation in RA, assays combining primary cells with total analyses of prostaglandins and leukotrienes can be used. The effects of DA are then determined using primary synovial fibroblasts from RA patients, after stimulation with TNF‐α and IL‐1β. Arachidonic‐acid metabolites are subsequently identified and quantified using LC‐MS/MS in a multiple reaction monitoring setting. This targeted metabolic analysis allows for rapid detection of a large number of bioactive lipids in a single experiment.

## In vivo models

The use of in vivo arthritis models is suitable as the first and final step of a bioassay‐guided project. These models are first used to validate clinical observations from traditional use and knowledge, and in the final step, to prove the efficacy of, for example, pure compounds. If the screening assays provide many hits, they may also be used to prioritise the purification and structural determination of bioactive molecules. There are several in vivo models of arthritis. For the in vivo studies of DA, the KRN (TCR transgenic mouse line from K/BxN line) T‐cell transfer in vivo model can be used. In this model, arthritis is initiated by autoantibodies targeting a ubiquitously expressed self protein, glucose‐6‐phosphate isomerase (GPI). The autoantibodies form immune complexes with the GPI on cartilage and synovial surfaces, leading to the activation of the downstream proinflammatory pathways predominant in RA, followed by the activation and recruitment of neutrophils and mast cells [76]. T cells from the mice expressing transgenic KRN TCRs are transferred to T‐cell deficient (TCRb‐/‐) non‐obese diabetic (NOD) mice expressing MHC II IAg7 [77]. The advantages of this model are the rapid onset of arthritis from day three after T‐cell transfer and the robust and reproducible development of arthritis in nearly 100% of T‐cell recipient mice. It allows the study of the effector phase of inflammatory arthritis after autoantibodies have been produced, which makes this an attractive model. Animal models should always be used with the aim to refine, restrict and reduce models and numbers, and follow ethical guidelines [78]. The approach described in this review aims to minimise the use of in vivo studies, but, as with any drug development project, in vivo evidence is ultimately required.

## Clinical trials

DA has gone through animal experiments and a pilot clinical observation study, which both indicated that the formulation could have potential in reducing the progression towards RA (unpublished results). Above, we have exemplified methods to unravel the antirheumatic effects and mechanisms of actions mediated by DA using phenotypic assays and animal models. To further explore the effect of DA in the prevention of RA and build clinical evidence of the medicine, standard clinical trials are required in humans. Thus, we propose a double‐blind, randomised placebo‐controlled trial, which targets ‘at‐risk’ individuals [79]. As previously described, ‘at‐risk’ individuals are characterised by ACPA positivity, joint symptoms involving arthralgia, stiffness and swelling, but not yet synovitis. The length of such a trial requires 1 year of treatment and another year for follow‐up.

In perspective, several classic TCM formulas have been used for centuries for the treatment of RA and are well established in the traditional clinical routine. According to the National Health Commission of the People's Republic of China, TCM medical services accounted for 14.5%–16.4% of the total medical services in 2019–2020, TCM professional (assistant) doctors accounted for 16.2%–16.5% of the total and 99% of community health service centres provided TCM services for patients by the end of 2020 [80]. Generally, about 10% of RA patients in remission or with low disease activity are treated with pure TCM in the outpatients' department in Guangdong Provincial Hospital of Chinese Medicine. Patients with moderate and high disease activity, are basically treated with integrated traditional Chinese and Western medicine.

However, there are a limited number of clinical trials and thus the evidence is low. The previous meta‐analysis demonstrated that TCM may target the immune system and help alleviate RA through different mechanisms—for instance, to lower the levels of both RF and anti‐cyclic citrullinated peptides (anti‐CCP) [81], to help protect the bones and cartilages and reduce damages [82] and to decrease the disease activity [83]. As outlined, treating the disease is not the only goal. TCM theories attach great importance to preventing the occurrence of diseases by changing lifestyle, for example by ceasing smoking, weight reduction and physical activity, in order to maintain good health [84]. This coincides with the emerging concept of actually being able to prevent RA for individuals at very high risk. So is there any previous evidence for the prevention of RA using TCM according to these standards? To date, at the start of our project, the answer is no. There is one ongoing clinical trial in China trying to investigate the potential of TCM in RA prevention to intervene in CCP or RF‐positive individuals for 24 weeks [85], using a formula called Huayu–Qiangshen–Tongbi decoction, which has been through both pharmacological investigation [86] and clinical studies [87] in RA treatments. However, the result has not been published yet.

## Discussion

The pharmacology of plant‐based medicine is complex, comprising a large number of targets and an even higher number of possible active compounds. This comes with challenges of showing efficacy data and mechanisms of actions. Herein we provide a strategy to deconvolute the activity of formulations, that is, decoctions and mixtures of plants, used in Chinese medicine for the treatment and prevention of RA. This disease is of particular interest because it is possible to identify individuals at very high risk of developing the disease using biomarkers, such as ACPA and other autoantibodies.

The strategy builds on bioassay‐guided isolation of active components of individual plants, using a set of pharmacological assays representing key pathways of disease pathology, followed by tests in RA models in vivo. The extraction and isolation protocol is adapted to a high content, medium‐throughput bioassay format, in which the capacity lies in the range of one or two microtitre well plates (2 × 96) per assay and day. Results from these assays provide the rationale to proceed to animal models (e.g., CIA and adopted cell transfer, arthritis models). Despite the pathway‐oriented analyses, the exact mechanism of action remains to be elucidated.

In this context, it is noteworthy that the exact mechanism is unknown for many of the drugs used today, including acetominophene, MTX and other conventional DMARDs, and indeed the antimalarial artemisinin despite decades of clinical use and research. Conventional ways of describing mechanisms of action include screenings of enzyme and receptor functions, and measurement of target (protein) binding using techniques such as surface plasmon resonance, fluorescence spectroscopy, calorimetry and thermoshift assays. Nevertheless, the complexity of the chemical composition of the starting material renders these types of late‐stage analyses not necessary for the understanding of the plant‐based drug itself. Rather, we emphasise the importance of pathway analyses, in vivo characterization and, subsequently, clinical trials.

Omic techniques have been used to elucidate mechanisms in the steps between pathway analyses and in vivo experiments. Some examples are particularly intriguing. For instance, metabolomic analyses of animal plasma and blood after oral treatment with traditional medicine has been used to narrow down the potential active compounds to those absorbed, and thus likely to mediate the effect. In analogy to method development to understand pharmacology, the trend in the field of natural product chemistry is shifting towards the use of big data–driven approaches for structural characterization. Examples include mass spectrometry databases such as Global Natural Product Social Molecular Networking [88] for mass spectrometry–based identification and dereplication, and Small Molecule Accurate Recognition Technology [89] to aid structural elucidation by NMR; as well as commercial databases for MS‐based identification of TCM in particular. We encourage the use of these tools, but it should be kept in mind that they are primarily developed to either (i) quickly identify previously described compounds, (ii) target specific compound classes or (iii) expedite the discovery of new compounds. While the discovery of new compounds is highly appreciated by natural product chemists, we think that a great deal of value lies in evaluating ‘old’ compounds for new activity. The discovery of a new compound from a natural source is usually only accompanied by a single biological assay to test its effectiveness, often selected based on what can be offered by the neighbouring laboratory.

In the work on a formula such as DA, it will not be surprising that the bioassay‐guided isolation often results in known compounds. Most plants in that formulation are well known and have been subject to numerous studies. However, the aims of such projects are twofold: (i) to provide an understanding of and evidence for or against the use of traditional medicines and (ii) to understand the chemical components that mediate activity. From those perspectives, known compounds are also of interest. In this context, it should be highlighted that despite numerous compounds reported from several of the DA plants, some classes of compounds are neglected in TCM plants. In our project, we are targeting one such class in particular, namely ribosomally produced peptides [90].

The presence of multiple active components and the questions of synergistic and/or additive effects are prominent in the deconvolution of activity of plant extracts. The fact that hits in early stages of separation (e.g., of crude extracts) may be difficult to assign to one single subfraction or one single compound is recognised by most natural product chemists. Removing or adding components, for example, spiking the sample with an identified active compound or inhibitors of certain pathways, may help to identify the main bioactive compound.

Drug discovery based on ethnopharmacology and traditional medicine has been met with skepticism—about clinical evidence and safety, but also regarding matters related to biopiracy, collection and cultivation. These issues need to be addressed in any drug discovery project based on natural products but should not deter research in the field. A substantial number of our current drugs are natural products or derivatives thereof, and without doubt nature will continue to be a source of future discoveries. Therefore, continuous research based on the traditional use of plants is highly motivated. In our opinion, the strategy of starting from knowledge in traditional medicine, followed by the combination of in vivo evidence of efficacy and bioassay‐guided isolation to understand the chemistry and pathways involved, is one effective way forward.

## Conflict of interest

The authors declare that they have no conflict of interest.

## Author contributions

Per‐Johan Jakobsson: Conceptualization; funding acquisition; investigation; methodology; project administration; resources; supervision; writing – original draft; writing – review and editing. Ulf Göransson: Conceptualization; funding acquisition; investigation; methodology; project administration; resources; supervision; writing – original draft; writing – review and editing. Wen Zehuai: Conceptualization; investigation; methodology; project administration; supervision; writig – original draft. Huang Runyue: Conceptualization; investigation; methodology; supervision; writing – original draft. Marina Korotkova: Conceptualization; investigation; methodology; supervision; writing – original draft. Luke Robertson: Conceptualization; investigation; methodology; supervision; visualization; writing – original draft. Janika Welzel: Conceptualization; investigation; methodology; visualization; writing – original draft. Mingshu Zhang: Conceptualization; investigation; methodology; writing – original draft. Yang Zhihua: Conceptualization; investigation; methodology; writing – original draft. Gao Kaixin: Conceptualization; investigation; methodology; writing – original draft.
